# Alterations of Chinese women’s skin microbiota associated with the aging process: Characteristics of Chinese women′s skin microbiome

**DOI:** 10.3724/abbs.2022105

**Published:** 2022-08-18

**Authors:** Caixia Wang, Suzhen Yang, Laiji Ma, Yan Li, Yanqin Shi, Man Wang, Li Shao, Zongjie Li, Yue Wang

**Affiliations:** 1 Shanghai Institute of Technology Shanghai 201418 China; 2 The Oriental Beauty Valley Research Institute Shanghai 201403 China; 3 Shandong Freda Biotech Co. Ltd Jinan Shandong 250101 China; 4 Shanghai Jiaotong University Affiliated Sixth People’s Hospital South Campus Shanghai 201499 China

Skin is frequently mentioned as the first defense line of the body, which can prevent the invading pathogens colonization and maintain the balance of skin microbiota [1]. The formation and maturation of skin microbiota can be impacted by both external factors (e.g., living environment, diet, weather, exposure to pollutants, *etc*.) and endogenous factors (e.g., race, age, gender, cellular metabolisms, immune activity, hormone condition, *etc*.). Recently, the role of the taxonomic compositions of skin microbial communities in skin health and disease and their relationships with the skin aging process are broadly focused on and further investigated [2]. Skin aging is mainly associated with the changes of cutaneous physiology and the compositions of skin microbial community. Cellular metabolisms, immune activity, and hormone condition can also influence the skin aging process [3].

Advances in the next generation sequencing techniques and bioinformatics have already promoted the investigations on the skin microbial communities, and much more specific functions of the human skin microbiome have been characterized [4]. The exterior interface of the human skin is characterized by the desiccated and harsh physical landscapes, therefore the nutrient-poor and acidic environment are proper for the diverse commensal microbiota to colonize [5]. Three predominant species of skin microbiome ( *Cutibacterium acnes*, *Staphylococcus epidermidis* and *Staphylococcus aureus*) on the human epithelia have been frequently concerned. The functions of the produced antimicrobials, short-chain fatty acids, and other metabolites and their interactions with the host immune system need to be further investigated. Recently, the relationship between skin microflora and skin health has been much focused on, especially in the field of scientific skin care research area. In fact, skin microbiota is closely related to skin physiological characteristics, therefore the compositions of skin microbiome can reflect the state of skin immune homeostasis [6]. Until now, very few studies have been conducted on the associations between the aging process and the skin microbiome. Therefore, it is of great importance to study the relationships among the skin microbiota, skin aging and skin physiological parameters. In the present study, we mainly investigated the microbial compositions of skin microbiome in Chinese women, and the relationship between the skin aging and the skin microbiota was also analyzed.

A total of 242 female volunteers living in Shanghai were sampled to determine the facial skin microbial communities. Participants were grouped by age: 114 young adults (26.2±2.3 y, W0 group), 105 middle-aged adults (32.6±2.8 y, W1 group) and 23 elderly adults (46.1±4.5 y, W2 group). The inclusion criteria included the following items: healthy adults with 18~50 years of age who live in Shanghai; being aware of the research purpose; read, understood and signed the informed consent forms. The exclusion criteria included the following items: received anti-inflammatory drugs or antibiotics orally or by injection within 3 months; facially applied anti-inflammatory drugs within 2 months; undergoing treatments for asthma or other chronic respiratory diseases; suffering from immunodeficiency or autoimmune diseases; lactating or pregnant women; volunteers already enrolled in other clinical traits. This research was carried out in accordance with the Declaration of Helsinki (2013 revised edition, www.wma.net/en/30publications/10policies/b3/index.html). Ethics approval was provided by the Institutional Review Board of Shanghai Fengxian Central Hospital (Approval No. 2021-KY-15).

Referring to the classification method of the Skin Aging Atlas Volume 2-Asian Type and facial wrinkle scale (FWS), the crow’s feet aging rating system based on the number and depth was performed on the human face. The laboratory condition was standardized under the guidance of the *Cosmetics Evaluation Guide*. The test temperature and relative air humidity were maintained at 21±1°C and 50%±10%, respectively. A Visia-CR facial image analyzer (Canfield Scientific Inc, Fairfield, USA) was used to take and save the photos of the front and the side of the volunteers’ faces. Sebumeter® SM 815 (Courage & Khazaka electronic GmbH, Cologne, Germany), Glossymeter® GL200 (Dpro Scientific, Petaling Jaya, Malaysia), Mexameter® MX 18 (Courage & Khazaka electronic GmbH), pH 905 (Courage & Khazaka electronic GmbH) and Cutometer dual MPA 580 (Courage & Khazaka electronic GmbH) were used to measure the sebum, glossiness, melanin, erythema, pH and elasticity of the corresponding area under the left and right zygomatic bone. Volunteers were required not to use any skin care products before sample collection, and all the volunteers were required to keep their faces un-washed on the sampling day in the morning. The sampling locations were set as 3× 3 cm on the left side of the cheek, the symmetrical right side, and the middle of the forehead. The relevant locations were wiped with three sterile swabs for at least 15 times, and then the swabs were put into sterile tubes and stored at –80°C.

A FastDNA® Spin Kit (Omega Bio-tek, Norcross, USA) was used to extract total DNA from the samples. The concentration and purity of DNA were assessed with NanoDrop 2000 (Thermo Fisher Scientific, Waltham, USA), and the integrity of DNA was detected by 1% agarose gel electrophoresis. Bacterial DNA was used as the template for amplifying the V3–V4 region of the 16S rRNA gene. The forward and reverse primers were 338F: 5′-ACTCCTACGGGAGGCAGCAG-3′ and 806R: 5′-GGACTACHVGGGTWTCTAAT-3′. The PCR reaction mixture (20 μL) included 4 μL FastPfu Buffer, 2 μL 2.5 mM dNTPs, 0.8 μL 5 μM Forward Primer, 0.8 μL 5 μM Reverse Primer, 0.4 μL FastPfu Polymerase, 0.2 μL BSA, and 10 ng template DNA. The PCR program began with a 3 min denaturation step at 95°C, followed by 27 cycles of 30 s at 95°C (denaturation), 30 s annealing at 55°C, and 30 s elongation at 72°C, followed by a final 10 min extension at 72°C. Each sample had a mixture of three PCR repeats, and 2% agarose gel electrophoresis was used to detect the amplified product. The purification of PCR products was carried out using the AxyPrep DNA Gel Extraction Kit (Axygen Biosciences, Union City, USA). After being quantified with Quantus™ Fluorometer (Promega, Madison, USA), the PCR products were mixed into corresponding proportions. The DNA sequencing library was constructed using the NextFlex Rapid DNA-Seq Kit (Bioo Scientific, Austin, USA), and then the purified PCR products were subjected to Illumina-based high-throughput sequencing on a MiSeq PE300 platform (Illumina, San Diego, USA) [7]. Raw fastq ( https://github.com/OpenGene/fastp, version 0.20.0) files were demultiplexed and quality-filtered using FLASH ( http://www.cbcb.umd.edu/software/flash, version 1.2.7). Operational Units (OTUs) were clustered with a 97% similarity cutoff using UPARSE ( http://drive5.com/uparse/, version 7.1). The phylogenetic affiliation of each 16S rRNA sequence was analyzed using RDP Classifier ( http://rdp.cme.msu.edu/, version 2.2) against the SILVA database (v138) with a confidence threshold of 70%. Alpha diversity analysis in richness (ACE and Chao indices) and diversity (Shannon and Simpson indices) was conducted using Mothur software ( https://www.mothur.org). The linear discriminant analysis effect size (LEfSe) was performed to search for the taxa of which the relative abundances were significantly different among the three groups. SPSS software version 25.0 (IBM Corp., Chicago, USA) was used for statistical analysis. According to the classification of wrinkles, the nonparametric test and Kruskal-Wallis test were used to analyze the skin physiological indices.

The degree of wrinkles and the skin physiological parameters were analyzed among the three different groups ( Supplementary Table S1 ). With the increase of wrinkles, there was an increasing trend of pH, but the difference did not reach statistical significance ( *P*>0.05). The levels of sebum showed a non-significant decreasing trend ( *P*>0.05), and the glossiness did not change significantly either ( *P*>0.05). However, the increase of melanin and erythema and the decrease of elasticity were found to be significantly different among the three groups ( *P*<0.01).

A total of 23,513,531 quality-filtered and chimera-checked sequences were obtained with an average length of 415 bp ( Supplementary Table S2 ). Clusters and annotations were performed on 242 samples at 97% similarity level, and a total of 16375 OTUs were obtained, belonging to 61 phyla, 2091 genera, and 4843 species. The richness and diversity of the skin microbial communities in different groups were evaluated using the alpha diversity indices, while the Chao1 and Ace indices were calculated for the richness analysis, and the Shannon and Simpson indices were calculated for the diversity analysis ( Figure 1A–D). The ACE and Chao1 indices indicated that the bacterial richness of W0 group was significantly lower than that of the W1 group, and the Shannon and Simpson indices revealed that the bacterial community diversity of W0 group was also significantly lower than those of the W1 and W2 groups. Therefore, the alpha diversity analysis revealed that the bacterial community richness and diversity were obviously lower in the young group (W0) than in the middle-aged group (W1) and the elderly group (W2). To assign the taxonomic composition of skin microbiota, the RDP classifier was used to compare the bacterial community structure at both phylum level and genus level ( Figure 1E,F). At the phylum level, the 5,100,634 bacterial sequences of the 242 samples belonged to *Actinobacteria* (4439584, 64.76%), *Proteobacteria* (1172946, 17.11%), *Firmicutes* (1009773, 14.73%), *Bacteroidetes* (102030, 1.49%) and other phyla (131285, 1.91%). At the genus level, the OTUs mainly belonged to *Cutibacterium* (4003868, 58.40%), *Staphylococcus* (643124, 9.38%), *Pseudomonas* (252851, 3.69%), *Rhodococcus* (167668, 2.45%), *Streptococcus* (153573, 2.24%), *Corynebacterium* (106488, 1.55%), *Oligotrophomonas* (87299, 1.27%), *Burkholderia* (74741, 1.09%), and other genera ( Supplementary Table S3 ).

**Figure 1:**
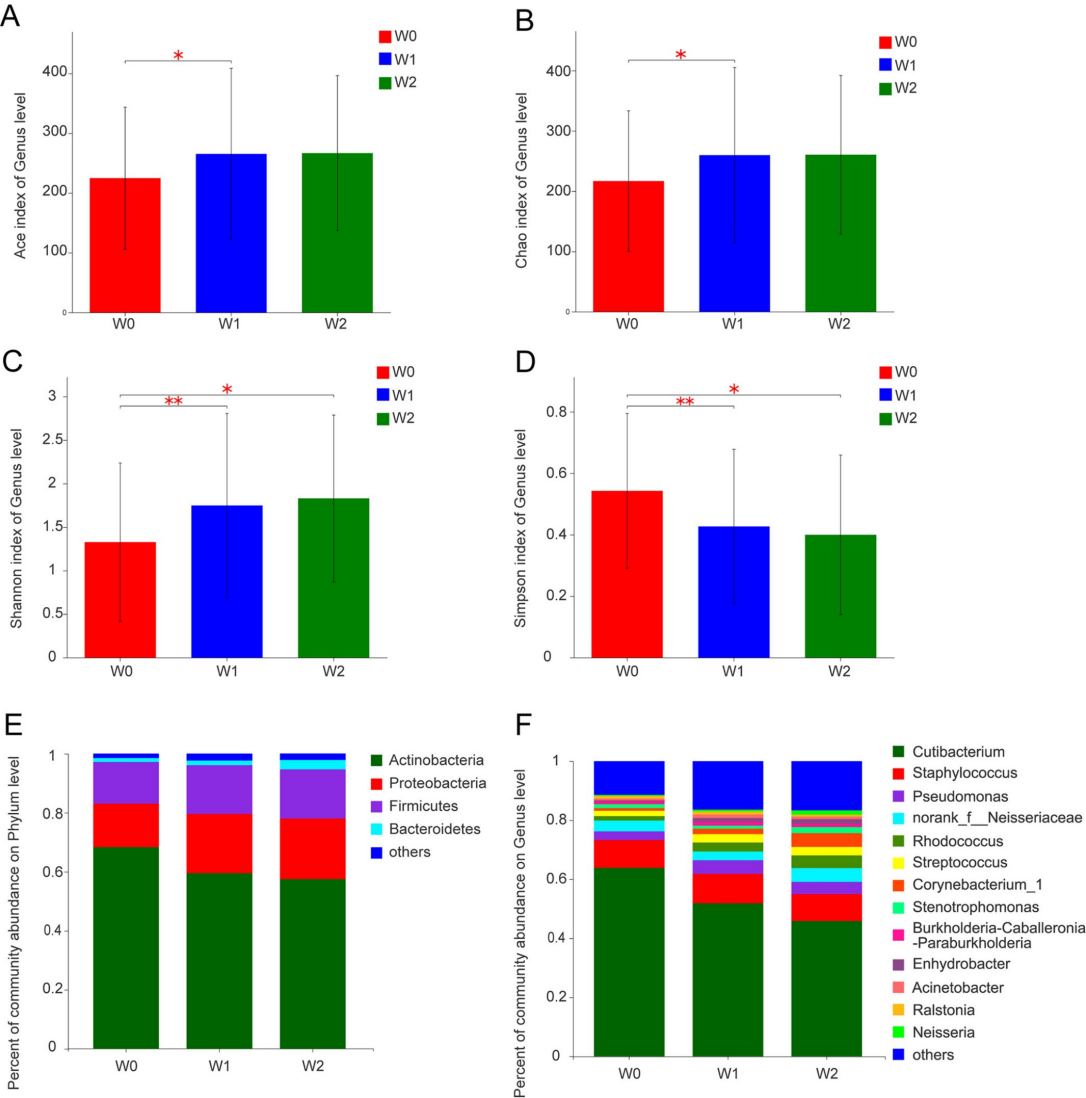
Figure 1 Alpha diversity analysis and the relative abundances of the skin microbiome at the phylum and genus levels The richness estimators of ACE (A) and Chao (B) and the diversity indices of Shannon (C) and Simpson (D) were calculated, respectively. The ACE and Chao1 indices indicated that the bacterial richness of the W0 group was significantly lower than those of the W1 and W2 group, while the Shannon and Simpson indices revealed that the bacterial community diversity of W0 group was also significantly lower than those of the W1 and W2 groups. Bacterial groups of the phylum (E) and genus (F) levels were analyzed and compared, and the taxa communities less than 1% abundance were merged into others.

After obtaining the data on community abundance, we used the Kruskal-Wallis H test to analyze the different microbial communities among the three groups. The portion of *Actinobacteria* in the W1 and W2 groups was significantly lower than that in the W0 group ( *P*=0.0054), while the portion of *Proteobacteria* was increased significantly ( *P*=0.0480). At the genus level, statistical analysis for the top 15 genera revealed that the relative abundances of *Cutibacterium* ( *P*=0.0007), *Corynebacterium* ( *P*=0.0002), *Streptococcus* ( *P*=0.04435), *Enhydrobacter* ( *P*=0.0170), *Neisseria* ( *P*=0.0153), *Chryseobacterium* ( *P*=0.0030), and *Acinetobacter* ( *P*=0.0071) were significantly different among the three groups ( Figure 2A).

**Figure 2:**
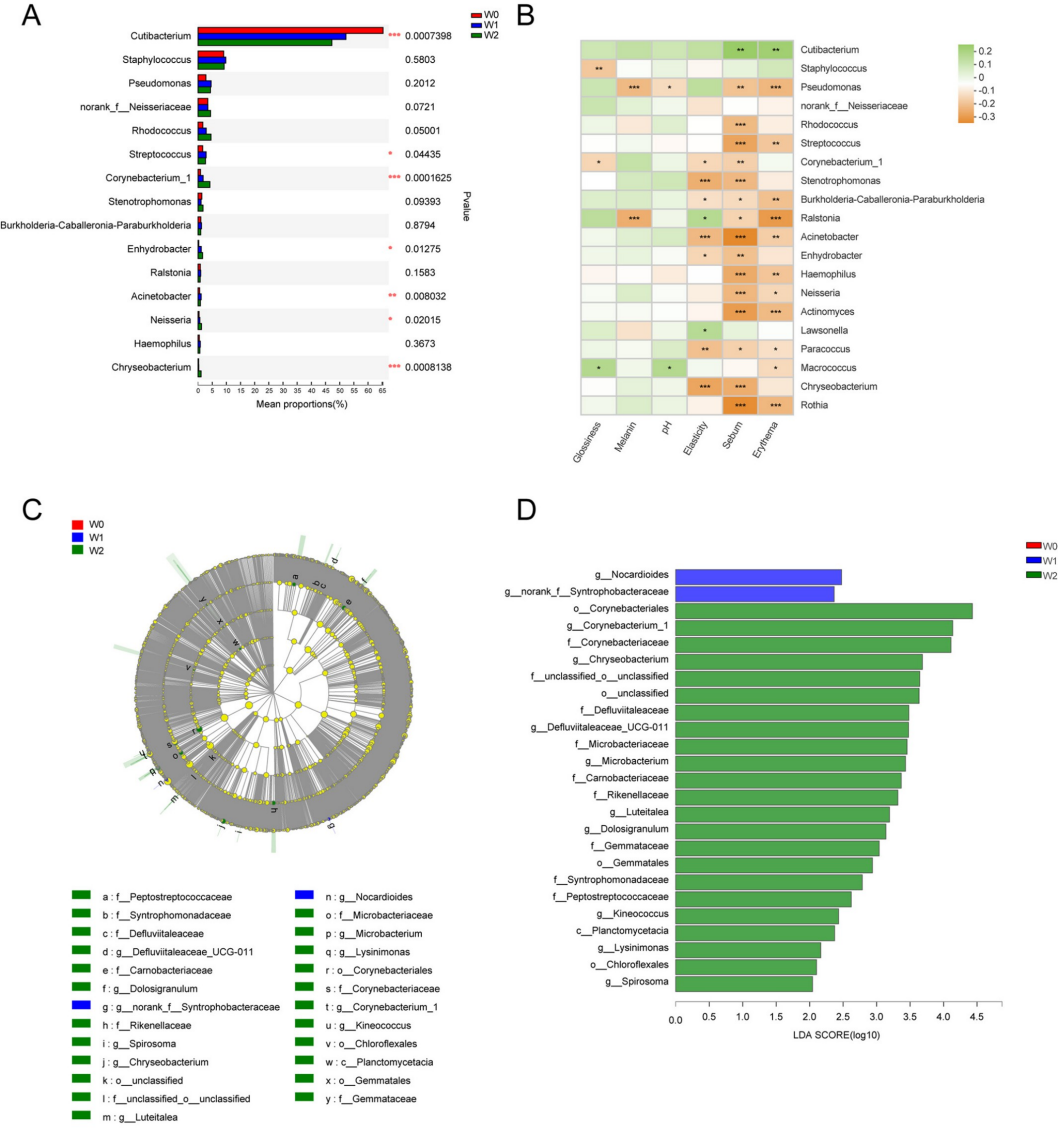
Figure 2 Comparisons of the differences in the bacterial relative abundances at the genus level, the spearman correlation heatmap and the LEfSe analysis The ordinate indicated the bacterial name at the genus levels, and the abscissa indicated the percentage values of the bacterial abundances (A). The correlation heatmap visualized the relationships between the skin microbiome and the skin physiological parameters (B). The R values were shown in different colors. The taxonomic cladogram identified the taxa with the greatest differences among the W0 group (red color), W1 group (blue color) and W2 group (green color), and the brightness of each dot was proportional to the effect size (C). The three groups with different LDA scores were indicated in different colors, and the taxa with a significant LDA threshold value of > 2 were shown (D).

Analysis of the top 20 genera and the six skin physiological parameters showed that there was a significant correlation between the changes of skin microbiota and skin physiological parameters ( Figure 2B). Sebum had a positive correlation with *Cutibacterium*. Erythema had a negative correlation with *Ralstonia*, *Actinnomyces* and *Pseudomonas*. Sebum had a negative correlation with *Rhodococcus*, *Neisseria*, *Streptococcus*, *Haemophilus*, *Actinnomyces*, *Stenotrophomonas*, *Acinetobacter* and *Chryseobacterium*. Elasticity had a negative correlation with *Stenotrophomonas*, *Acinetobacter*, *Paracoccus* and *Chryseobacterium*. Melanin had a negative correlation with *Pseudomonas* and *Ralstonia*. Linear discriminant analysis effect size (LEfSe) was conducted to demonstrate the differences of OTUs among the three groups ( Figure 2C,D). The taxonomic cladogram identified the taxa with the greatest differences in abundance among the W0 group (red color), W1 group (blue color) and W2 group (green color), and the brightness of each dot was proportional to the effect size. The three groups with different LDA scores were indicated in different colors, and the taxa with a significant LDA threshold value of >2 were shown.

Consistent with the previous studies, the skin physiological state and immune function of the elderly group decreased significantly, and the relative abundances of beneficial bacteria also decreased significantly [8]. We found that the skin of the elderly colonized much more opportunistic pathogens such as *Proteobacteria*, which resulted in higher diversity and abundance of microorganisms. At the genus level, the analyzed data showed that *Cutibacterium* was the most dominant genera of skin bacteria, and the relative abundance of which decreased significantly with the aging process ( Figure 1F). Because the immune function of the human skin decreased obviously during with the aging process, the colonization chance of certain types of opportunistic pathogens (such as *Corynebacterium*) increased [9]. In addition, our data also showed that there were also significant differences in the relative abundance of *Enhydrobacter* ( *P*=0.0170), *Acinetobacter* ( *P*=0.0071), *Neisseria* ( *P*=0.0153), and *Chryseobacterium* ( *P*=0.0030) during the aging process, which indicated that the skin bacterial communities had close relations with the skin aging.

The physiological changes of the skin could affect the composition and structure of skin microorganisms, which in turn further could promote the skin aging process. Generally, *Cutibacterium* and *Staphylococcus* are considered to be the sentinel skin microbiota which can maintain the skin homeostasis [10]. We analyzed the relationship between the skin physiological parameters and the microbial flora through correlation analysis of environmental factors by Spearman correlation analysis. Our data confirmed that *Corynebacterium* prefers a humid environment, therefore the relative abundance of *Corynebacterium* increased with the skin aging process ( Figure 2B). Among the top 20 genera , *Cutibacterium* was the only one that had a significant positive correlation with the skin sebum, while most of other bacteria had a negative correlation.

In conclusion, the correlation of skin physiological indices and skin microorganisms during aging process was investigated in the current study. Characteristics and alterations of Chinese women’s skin microbiota may have important value for the investigation and application of national skin-care and cosmetic products.

## Supplementary Data

Supplementary data is available at *Acta Biochimica et Biophysica Sinica* online.
